# Cholesterol Content of Very-Low-Density Lipoproteins Is Associated with 1-Year Mortality in Acute Heart Failure Patients

**DOI:** 10.3390/biom12101542

**Published:** 2022-10-21

**Authors:** Vesna Degoricija, Iva Klobučar, Ines Potočnjak, Sanda Dokoza Terešak, Luka Vidović, Gudrun Pregartner, Andrea Berghold, Hansjörg Habisch, Tobias Madl, Saša Frank

**Affiliations:** 1 School of Medicine, University of Zagreb, 10000 Zagreb, Croatia; 2 Department of Medicine, Sisters of Charity University Hospital Centre, 10000 Zagreb, Croatia; 3 Department of Cardiology, Sisters of Charity University Hospital Centre, 10000 Zagreb, Croatia; 4 Institute for Clinical Medical Research and Education, Sisters of Charity University Hospital Centre, 10000 Zagreb, Croatia; 5 Department of Emergency Medicine, Sisters of Charity University Hospital Centre, 10000 Zagreb, Croatia; 6 Institute for Medical Informatics, Statistics und Documentation, Medical University of Graz, 8036 Graz, Austria; 7 Gottfried Schatz Research Center, Molecular Biology and Biochemistry, Medical University of Graz, 8010 Graz, Austria; 8 BioTechMed-Graz, 8010 Graz, Austria

**Keywords:** acute heart failure, very-low-density lipoprotein, mortality, NMR spectroscopy, prognostic biomarkers, risk, cholesterol

## Abstract

Considering the relationship between the extent of metabolic derangement and the disease severity in heart failure, we hypothesized that the lipid content of very-low-density lipoprotein (VLDL) may have prognostic value for 1 year mortality in acute heart failure (AHF). Baseline serum levels of VLDL cholesterol (VLDL-C), VLDL triglycerides (VLDL-TG), VLDL phospholipids (VLDL-PL), and VLDL apolipoprotein B (VLDL-apoB) were measured using NMR spectroscopy. We calculated the ratios of the respective VLDL lipids and VLDL apoB (VLDL-C/VLDL-apoB, VLDL-TG/VLDL-apoB, and VLDL-PL/VLDL-apoB), as estimators of the cholesterol, triglyceride, and phospholipid content of VLDL particles and tested their association with mortality. Out of 315 AHF patients, 118 (37.5%) patients died within 1 year after hospitalization for AHF. Univariable Cox regression analyses revealed a significant inverse association of VLDL-C/VLDL-apoB (hazard ratio (HR) 0.43, 95% confidence interval (CI) 0.29–0.64, *p* < 0.001), VLDL-TG/VLDL-apoB (HR 0.79, 95% CI 0.71–0.88, *p* < 0.001), and VLDL-PL/VLDL-apoB (HR 0.37, 95% CI 0.25–0.56, *p* < 0.001) with 1 year mortality. Of the tested parameters, only VLDL-C/VLDL-apoB remained significant after adjustment for age and sex, as well as other clinical and laboratory parameters that showed a significant association with 1 year mortality in the univariable analyses. We conclude that cholesterol content of circulating VLDL (VLDL-C/VLDL-apoB) might be of prognostic value in AHF.

## 1. Introduction

Heart failure (HF) is a final stage of various cardiovascular diseases and, therefore, a frequent cause of disability and death worldwide [1]. The failing heart exhibits an altered structure and consequently impaired function, resulting in a diminished perfusion of the metabolizing tissues. Acute heart failure (AHF) is characterized by either the rapid onset or worsening of the signs and symptoms of HF [2]. 

Neurohormonal activation triggered by left-ventricular dysfunction and consequent tissue hypoperfusion, together with tissue congestion due to right-sided HF, increase serum levels of catecholamines, natriuretic peptides, and inflammatory cytokines [3,4]. These promote catabolic dominance, the hallmark of metabolic dysfunction in HF [5]. This unfavorable state in HF patients is further deteriorated by an impaired intestinal nutrient absorption due to congestion-induced intestinal edema, a reduced appetite, and the diminished biosynthetic capacity of the hypo-perfused and/or congested liver, as well as a progressive worsening of renal function [3,5,6,7]. 

Very-low-density lipoprotein (VLDL), a lipoprotein secreted by the liver is the principal carrier of triglycerides (TG) in human serum and a precursor of low-density lipoprotein (LDL) [8]. In addition to triglycerides, being the predominant lipids, VLDL particles contain cholesterol (C) and phospholipids (PL), as well as different apolipoproteins including one molecule of apolipoprotein B-100 (apoB) [9,10,11]. Several factors affect lipid content and secretion rate of VLDL. These include substrate availability for the de novo lipogenesis and protein biosynthesis, insulin sensitivity, and hepatic TG content, as well as the activity of the molecular machinery involved in the VLDL assembly and secretion [10,12,13]. While regular feeding and efficient intestinal absorption and delivery of nutrients to the liver ensure substrate availability, the assembly and secretion of VLDL are largely dependent on the overall liver fitness, known to be frequently impaired in HF patients [5,14]. Upon secretion, circulating VLDL particles undergo progressive processing mediated by serum lipases and neutral lipid transfer/exchange proteins, resulting in the depletion of TG and PL and concomitant conversion of VLDL to intermediate-density lipoprotein (IDL) and LDL [9,15,16].

The estimation of risk based on established multivariable predictive models (comprising various serum biomarkers, as well as patients’ clinical characteristics and clinical signs of the disease) is not adequate, and the mortality rate in AHF patients remains unacceptably high [17,18]. Therefore, adding newly identified serum biomarkers to the existing panel of the currently utilized prognostic serum biomarkers may improve the performance of predictive models and, thus, help physicians in initiating appropriate therapeutic interventions and improve the outcome of AHF patients [19].

While a couple of studies reported the association of LDL-cholesterol levels with mortality in patients with HF [20,21,22], the association of VLDL with mortality in AHF has not been examined so far. Considering catabolic dominance and impaired hepatic biosynthetic activity in HF, we assumed that the lipid content of VLDL particles is affected by the disease severity and associated with mortality in AHF patients. Here, we show that low C (but not TG or PL) content of VLDL is associated with increased mortality in AHF patients and might, thus, be of prognostic value in AHF. 

## 2. Materials and Methods

### 2.1. Study Design and Patients

Study design, inclusion and exclusion criteria, and patient characteristics for the AHF cohort were described in our previous study [23] and are shown in Scheme S1. The AHF study was a prospective, observational study including consecutive patients who were hospitalized due to AHF. The diagnosis of AHF and the treatment of all patients were according to the definition and guidelines given by the European Society of Cardiology [2]. Written informed consent was obtained from each enrolled patient in compliance with Good Clinical Practice Guidelines, and the study was conducted in adherence to the principles of the Declaration of Helsinki [24]. The study was approved by the local Ethics Committees of the Sisters of Charity University Hospital Center, Zagreb, Croatia (EP 2258/18-10) and the Medical University of Graz, Austria (EK 33-258 ex 20/21). Participants were followed up every 3 months for 1 year. The primary endpoint was all-cause mortality after 1 year.

### 2.2. Laboratory Procedures

The collection of the blood samples and the standard laboratory methods were described in our previous report [23]. 

### 2.3. Lipoprotein Profiling by Nuclear Magnetic Resonance (NMR) Spectroscopy

Blood serum lipoproteins were measured on a Bruker 600 MHz Avance Neo NMR spectrometer using the Bruker IVDr lipoprotein subclass analysis protocol as described [25]. Briefly, serum samples were thawed, and 330 µL of each sample was mixed with 330 µL of Bruker serum buffer (Bruker, Rheinstetten, Germany). The samples were mixed gently, and 600 µL of the mixed sample was transferred into a 5 mm SampleJet rack tube (Bruker, Rheinstetten, Germany). Proton spectra were obtained at a constant temperature of 310 K using a standard nuclear Overhauser effect spectroscopy (NOESY) pulse sequence (Bruker, Rheinstetten, Germany: noesygppr1d), a Carr–Purcedll–Meiboom–Gill (CPMG) pulse sequence with presaturation during the relaxation delay (Bruker, Rheinstetten, Germany: cpmgpr1d) to achieve water suppression, and a standard 2D J-resolved (JRES) pulse sequence (Bruker, Rheinstetten, Germany: jresgpprqf). Data analysis was carried out using the Bruker IVDr LIpoprotein Subclass Analysis (B.I.LISA^™^, Rheinstetten, Germany) method. 

### 2.4. Statistics

Metric parameters are summarized as the mean and standard deviation or median and interquartile range (q1, q3), whereas absolute and relative frequencies are used to describe categorical parameters. Differences in patients who survived and those who died within 1 year, as well as between groups defined by various clinical characteristics, were tested using the t-test, Mann-Whitney U test, or Fisher’s exact test. The Spearman correlation coefficient was used to assess correlations between the estimators of VLDL lipid content (ratios of VLDL-lipids (mg/dL) and VLDL-apoB (mg/dL): VLDL-C/VLDL-apoB, VLDL-TG/VLDL-apoB, and VLDL-PL/VLDL-apoB) and various clinical and laboratory parameters. Univariable and multivariable Cox regression analyses were used to examine the prognostic value of VLDL-C/VLDL-apoB, VLDL-TG/VLDL-apoB, and VLDL-PL/VLDL-apoB for 1 year mortality. In the multivariable analyses, we adjusted for age, sex, body mass index (BMI), mean arterial pressure (MAP), estimated glomerular filtration rate (eGFR), blood urea nitrogen (BUN), C-reactive protein (CRP), N-terminal pro brain natriuretic peptide (NT-proBNP), hemoglobin, alanine aminotransferase (ALT), albumin, and total cholesterol. Results are presented as the hazard ratio (HR) and the respective 95% confidence interval (CI). A *p*-value <0.05 was generally considered significant, except for the correlation analyses of VLDL-C/VLDL-apoB, VLDL-TG/VLDL-apoB, and VLDL-PL/VLDL-apoB, as well as for the differences in these ratios among various groups of AHF patients, where a Bonferroni correction was applied to correct for multiple testing and, thus, a *p*-value <0.017 was considered significant. R version 4.1.0 was used for these analyses. 

## 3. Results

### 3.1. Clinical Characteristics, Medication, and Laboratory Parameters 

Baseline clinical characteristics, medication, and laboratory parameters of the whole cohort were described in our previous report [23] and are shown in Table 1 and Table S1 as a comparison between the patients who were alive and those who died within 1 year after hospitalization for AHF. The serum samples of 315 hospitalized patients with AHF that completed the 1 year follow-up were used for the present analyses. Of these, 118 (37.5%) patients died within 1 year after hospitalization for AHF. 

### 3.2. Serum Levels and Lipid Content of VLDL Parameters

To determine the lipid content of VLDL particles, we measured baseline serum levels of VLDL-C, VLDL-PL, VLDL-TG, and VLDL-apoB using NMR spectroscopy (Table 2). Of note, the apoB content of VLDL is proportional to the VLDL particle number as each VLDL contains exactly one molecule of apoB [9]. This is illustrated by a perfect correlation between VLDL-apoB and VLDL-particle concentrations determined by NMR in the present study (*r* = 1, *p* < 0.001). Considering this, we calculated the ratios of the respective VLDL-lipids and VLDL-apoB (VLDL-C/VLDL-apoB, VLDL-TG/VLDL-apoB, and VLDL-PL/VLDL-apoB), as estimators of the C, TG, and PL content of VLDL particles. As shown in Table 2, the ratios were significantly lower in the patients who died compared to those who were alive 1 year after hospitalization for AHF, indicating impaired lipidation or increased de-lipidation of VLDL in these patients. One patient who died within 1 year after hospitalization for AHF had no available data to calculate these ratios, and, in one patient who was alive 1 year after hospitalization for AHF, the VLDL-TG value was below the detection limit of NMR.

### 3.3. Correlation Analyses of VLDL-C/VLDL-apoB, VLDL-TG/VLDL-apoB, and VLDL-PL/VLDL-apoB Ratios with Clinical and Laboratory Parameters

**Figure 1:**
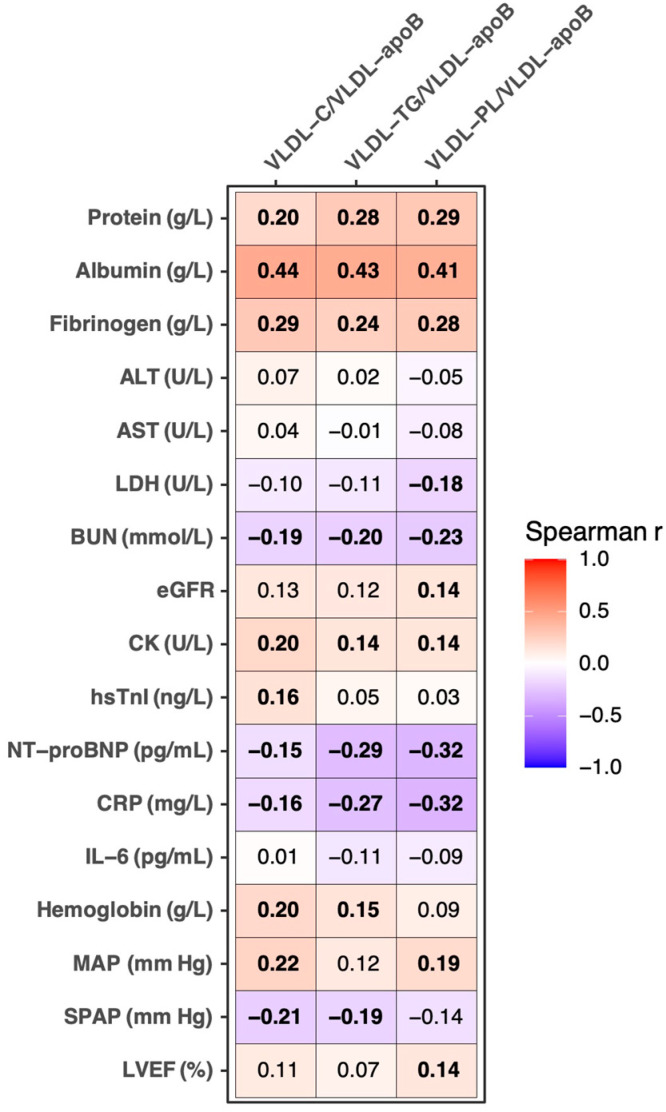
Heatmap for correlations between the ratios estimating VLDL lipid content and laboratory and clinical parameters. Values presented are the Spearman correlation coefficients. The *p*-values <0.017 are considered significant after a Bonferroni correction for multiple testing and significant correlations are depicted in bold. SPAP was measured in 259 patients; otherwise, the analyses are based on 314 samples for VLDL-C/VLDL-apoB and VLDL-PL/VLDL-apoB, respectively, and 313 for VLDL-TG/VLDL-apoB. BUN, blood urea nitrogen; CK, creatine kinase; CRP, C-reactive protein; eGFR, estimated glomerular filtration rate; hsTnI, high-sensitivity troponin I; LDH, lactate dehydrogenase; MAP, mean arterial pressure; NT-proBNP, N-terminal pro brain natriuretic peptide; SPAP, systolic pulmonary pressure.

After applying a Bonferroni correction for testing multiple parameters, VLDL-C/VLDL-apoB, VLDL-TG/VLDL-apoB, and VLDL-PL/VLDL-apoB were significantly (*p* < 0.017) positively correlated with total serum protein, albumin, fibrinogen, and CK, as well as significantly negatively with BUN, NT-proBNP, and CRP (Figure 1). Furthermore, while both VLDL-C/VLDL-apoB and VLDL-TG/VLDL-apoB were significantly positively correlated with hemoglobin and significantly negatively correlated with systolic pulmonary artery pressure, both VLDL-C/VLDL-apoB and VLDL-PL/VLDL-apoB were significantly positively correlated with MAP. Additionally, VLDL-C/VLDL-apoB was significantly positively correlated with hsTnI, whereas VLDL-PL/VLDL-apoB was significantly positively correlated with eGFR and LVEF, as well as negatively with LDH. Neither ratio was significantly correlated with ALT, AST, or IL-6 (Figure 1).

### 3.4. VLDL-C/VLDL-apoB, VLDL-TG/VLDL-apoB, and VLDL-PL/VLDL-apoB Ratios in Various Groups of AHF Patients

While VLDL-C/VLDL-apoB was significantly higher in AHF patients with T2D, VLDL-TG/VLDL-apoB was significantly higher in AHF patients with CAD, as well as in AHF patients on statins. VLDL-C/VLDL-apoB, VLDL-TG/VLDL-apoB, and VLDL-PL/VLDL-apoB were significantly lower in AHF patients with signs of venous volume overload and in patients with AF, as well as in patients with AHF following CHF compared to new onset AHF cases (Table 3).

### 3.5. Association of the Estimators of VLDL Lipid Content with Mortality in AHF Patients

**Figure 2:**
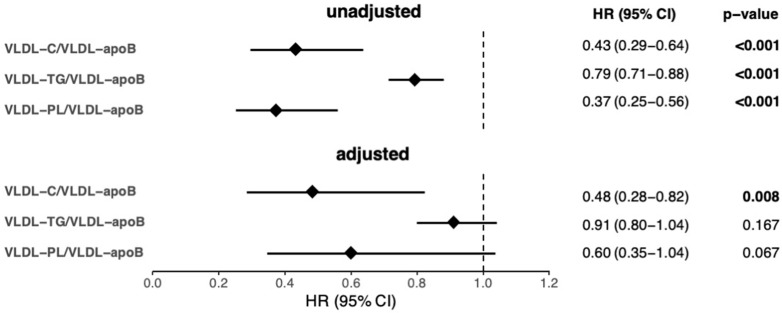
Cox regression analyses of the ratios estimating VLDL lipid content as predictors of 1 year mortality in AHF patients. In the adjusted model age, sex, BMI, MAP, eGFR, BUN, CRP, NT-proBNP, hemoglobin, ALT, albumin, and total cholesterol were used as covariates. For VLDL-C/VLDL-apoB and VLDL-PL/VLDL-apoB, the total number of patients and events in the univariable analyses was 314 and 117, respectively, and, in the adjusted analyses, these numbers were 302 and 111, respectively. For VLDL-TG/VLDL-apoB, the total number of patients and events in the univariable analyses was 313 and 117, respectively, and, in the adjusted analyses, these numbers were 301 and 111, respectively. Significant associations (*p* < 0.05) are depicted in bold. AHF, acute heart failure; apoB, apolipoprotein B; BMI, body mass index; BUN, blood urea nitrogen; C, cholesterol; CI, confidence interval; CRP, C-reactive protein; eGFR, estimated glomerular filtration rate; HR, hazard ratio; MAP, mean arterial pressure; NT-proBNP, N-terminal pro brain natriuretic peptide; PL, phospholipids; TG, triglycerides; VLDL, very-low-density lipoprotein.

VLDL-C/VLDL-apoB, VLDL-TG/VLDL-apoB, and VLDL-PL/VLDL-apoB were significantly associated with 1 year mortality in the univariable Cox regression analyses. However, only the association of VLDL-C/VLDL-apoB remained significant after adjustment for age, sex, and the clinical and laboratory parameters significantly associated with 1 year mortality in the univariable analyses shown in Table S2 (Figure 2). 

## 4. Discussion

The estimation of risk in AHF is difficult and associated with poor outcome of the patients [17,18]. New biomarkers, reflecting different aspects of the complex underlying AHF pathophysiology, may improve risk assessment and improve the overall management of AHF patients [19]. Previously, we and others identified the prognostic capacity of small HDL-particles for AHF and CHF mortality [26,27]. More recently, we reported on the robust prognostic value of high-density lipoprotein apolipoprotein A-II (HDL-apoA-II) in AHF patients [23]. In the present study, we demonstrate for the first time the association of low cholesterol content of circulating VLDL (VLDL-C/VLDL-apoB) with 1 year mortality in AHF patients. 

Cholesterol (free cholesterol and cholesterol ester) incorporation into VLDL occurs in hepatocytes during the assembly of the particle, as well as after VLDL secretion in a process mediated by cholesterol ester transfer protein (CETP), a serum transfer protein secreted by the liver, which exchanges TG of VLDL for cholesterol ester of HDL [10,28]. The amount of hepatic cholesterol available for incorporation into VLDL is critically dependent on the supply of dietary cholesterol to the liver via chylomicron remnants, as well as the rate of hepatic cholesterol biosynthesis [10]. Additionally, the biosynthetic activity of the liver also determines serum CETP levels and activity, which were found to be decreased in HF patients and negatively associated with HF severity [29]. Accordingly, in the present study, a more severe AHF pathophysiology with impaired liver biosynthetic activity due to hypoperfusion and congestion, impaired nutrient absorption (due to intestinal congestion), inflammation, and catabolic dominance might be a likely cause for the lower VLDL-C/VLDL-apoB in patients who died compared to those who were alive within 1 year after index episode of AHF. Indeed, we observed a positive correlation between VLDL-C/VLDL-apoB and the serum levels of albumin, a biomarker of biosynthetic capacity of the liver, as well as CK, whose decreased serum levels are related to a poor nutritional state, catabolic dominance, and muscle wasting, which are typical signs of advanced HF [30].

In the present study, VLDL-TG/VLDL-apoB and VLDL-PL/VLDL-apoB were also lower in patients who died compared to those who were alive. However, VLDL-TG/VLDL-apoB and VLDL-PL/VLDL-apoB were not associated with mortality after accounting for patient characteristics and laboratory parameters. As mentioned before, the cholesterol content of circulating VLDL represents a sum of the cholesterol incorporated into the particle within the liver and of cholesterol provided by CETP. Hence, the cholesterol content of VLDL increases after VLDL secretion. In contrast, the TG and PL content of the secreted VLDL decreases due to progressive degradation of TG and PL by lipoprotein lipase (LPL) and endothelial lipase (EL), respectively [31,32]. The LPL and EL activities undergo complex and multifactorial regulation by nutritional state, hormones, and inflammation, as well as endogenous protein inhibitors secreted by the liver [32]. Therefore, it is conceivable that neurohormonal activation, inflammation, insulin resistance, and congestion, the hallmarks of the HF pathophysiology, modulate the TG and PL content of circulating VLDL through modulation of LPL and EL activity. Accordingly, a combination of higher LPL and EL activity and lower hepatic lipidation is a likely cause for lower VLDL-TG/VLDL-apoB and VLDL-PL/VLDL-apoB in patients who died compared to those who were alive within 1 year after index episode of AHF. It is, therefore, conceivable that a strong modulation of VLDL (TG and PL) lipidation and de-lipidation by the underlying AHF pathophysiology, illustrated by a significant correlation of VLDL-TG/VLDL-apoB and VLDL-PL/VLDL-apoB with biomarkers of AHF severity (NT-proBNP) and inflammation (CRP), confounds the association of VLDL-TG/VLDL-apoB and VLDL-PL/VLDL-apoB with AHF mortality.

Interestingly, in contrast to the VLDL lipid content, the VLDL particle number (VLDL-apoB) was similar in patients who died and those who were alive (Table 2) and was not correlated with biomarkers of HF severity, liver biosynthetic activity (with exception of fibrinogen), or inflammation (Table S3). This indicates that, in our AHF cohort, the lipid content better reflects the severity of the underlying AHF pathophysiology than the particle concentrations of VLDL. Along these lines, the estimators of the VLDL lipid content (VLDL-C/VLDL-apoB, VLDL-TG/VLDL-apoB, and VLDL-PL/VLDL-apoB) were lower in patients with a more severe AHF pathophysiology, i.e., those with signs of venous volume overload or atrial fibrillation, compared to those without, as well as in patients with AHF following CHF compared to the new onset AHF cases. This highlights the link between the VLDL lipid content and the patients’ overall state, as well as disease chronicity and severity [33,34].

Although a recent study identified VLDL-C as a risk factor for myocardial infarction [35], in the present study, the higher VLDL-C/VLDL-apoB improved outcome in patients with AHF, which is in line with a reverse epidemiology [36]. The question arises as to whether VLDL-C/VLDL-apoB is only a marker of the disease severity or an active player capable of counteracting detrimental effects of the AHF pathophysiology on various tissues, including a failing heart. 

There are several limitations to the present study. The design of the study precluded examination of the causality for the relationship between VLDL and other parameters. Therefore, the mechanistic relationship between lipid content of VLDL and the underlying pathophysiological processes could not be addressed. Additionally, since we determined cholesterol content of VLDL only on hospital admission, the impact of a therapeutic intervention, as well as any temporal development, could not be examined. We wish to point out that sampling, storage, and handling (freezing/thawing and aliquoting) of serum may have changed the concentrations of the VLDL parameters. Given the fact that, in the present study, the serum handling procedures were performed uniformly for each single sample according to the standardized protocols, the concentrations of the VLDL parameters were most likely affected to a similar extent in all serum samples. Accordingly, the serum handling procedures most likely only negligibly affected the relationship between the VLDL parameters and other clinical and laboratory parameters or the association of the VLDL parameters with mortality. Furthermore, since the patients’ nutritional state on admission was unknown, we could not assess the impact of fasting/feeding on the studied parameters. Considering the rather moderate number of available samples (*N* = 315), our results still need to be confirmed in larger AHF cohorts. 

## 5. Conclusions

On the basis of our results, we conclude that low baseline VLDL-C/VLDL-apoB is associated with increased 1 year mortality in AHF patients and might, thus, be of prognostic value in AHF.

## Figures and Tables

**Table 1:** Baseline characteristics and laboratory data of AHF patients upon hospital admission.

	Alive (*n* = 197)	Deceased (*n* = 118)	All (*n* = 315)	*p*-Value
**Demographics**				
Age (years)	72.5 (10.4)	77.0 (10.1)	74.2 (10.5)	**<0.001**
Sex, Female	85 (43.1%)	51 (43.2%)	136 (43.2%)	1.000
**Comorbidities**				
Hypertension	186 (94.4%)	108 (91.5%)	294 (93.3%)	0.355
T2DM	76 (38.6%)	56 (47.5%)	132 (41.9%)	0.127
CAD	100 (50.8%)	56 (47.5%)	156 (49.5%)	0.642
CMP	173 (87.8%)	115 (97.5%)	288 (91.4%)	**0.003**
AF	98 (49.7%)	72 (61.0%)	170 (54.0%)	0.062
CKD	72 (36.5%)	71 (60.2%)	143 (45.4%)	**<0.001**
MetS	130 (66.0%)	87 (73.7%)	217 (68.9%)	0.168
**Physical measures at admission**				
MAP (mmHg)	108.1 (24.2)	96.0 (19.5)	103.6 (23.3)	**<0.001**
Heart rate (beats/min)	103.8 (25.8)	95.3 (27.5)	100.6 (26.7)	**0.006**
Respiratory rate (breaths/min)	29.3 (6.9)	28.8 (6.0)	29.1 (6.5)	0.474
BMI (kg/m^2^)	27.4 (24.9, 30.7)	29.1 (25.3, 32.8)	28.0 (25.0, 31.6)	0.067
**Signs and symptoms**				
Symptom duration (days)	5.0 (3.0, 5.0)	5.0 (4.0, 5.0)	5.0 (4.0, 5.0)	**0.022**
Rales or crackles	193 (98.0%)	118 (100.0%)	311 (98.7%)	0.301
JVD	97 (49.2%)	77 (65.3%)	174 (55.2%)	**0.007**
Enlarged liver	95 (48.2%)	81 (68.6%)	176 (55.9%)	**<0.001**
Ascites	20 (10.2%)	29 (24.6%)	49 (15.6%)	**0.001**
Peripheral edema	114 (57.9%)	90 (76.3%)	204 (64.8%)	**<0.001**
**NYHA class**				0.305
3	13 (6.6%)	4 (3.4%)	17 (5.4%)	
4	184 (93.4%)	114 (96.6%)	298 (94.6%)	
**AHF type**				**0.003**
New onset AHF	24 (12.2%)	3 (2.5)%	27 (8.6%)	
AHF following CHF	173 (87.8%)	115 (97.5%)	288 (91.4%)	
**Echocardiography**				
LVEDd/BSA (mm/m^2^)	29.1 (4.9)	28.5 (5.2)	28.8 (5.0)	0.346
LVEF (%)	40.1 (11.9)	39.1 (12.6)	39.8 (12.1)	0.455
SPAP (mmHg)	47.0 (42.0, 55.0)	50.0 (45.0, 60.0)	50.0 (45.0, 60.0)	**0.005**
**AHF class**				0.575
HFrEF, EF <40%	88 (44.9%)	55 (51.4%)	143 (47.2%)	
HFmrEF, EF 41–49%	55 (28.1%)	26 (24.3%)	81 (26.7%)	
HFpEF, EF ≥50%	53 (27.3%)	26 (24.3%)	79 (26.1%)	
**Laboratory test results at admission**				
TC (mg/dL)	145.0 (118.0, 188.0)	128.5 (103.2, 156.8)	134.0 (110.0, 173.0)	**<0.001**
HDL-C (mg/dL)	43.0 (35.0, 52.0)	41.0 (31.0, 50.0)	42.0 (34.0, 51.0)	**0.022**
LDL-C (mg/dL)	78.0 (57.8, 109.2)	66.5 (49.2, 91.5)	73.0 (54.0, 102.0)	**<0.001**
Triglycerides (mg/dL)	90.0 (69.0, 121.0)	84.0 (68.0, 103.8)	88.0 (69.0, 113.5)	0.099
ApoB (mg/dL)	77.8 (65.6, 97.8)	75.7 (62.7, 89.9)	77.6 (63.8, 95.0)	**0.045**
Albumin (g/L)	38.2 (35.5, 42.0)	36.7 (33.8, 39.7)	37.8 (34.8, 41.3)	**0.009**
Total proteins (g/L)	67.0 (62.0, 72.0)	65.5 (61.0, 70.0)	67.0 (61.0, 72.0)	0.214
Bilirubin (µmol/L)	17.4 (11.0, 28.5)	17.2 (11.9, 29.2)	17.3 (11.1, 28.7)	0.336
AST (U/L)	28.0 (22.0, 42.0)	27.0 (18.2, 52.5)	28.0 (20.0, 44.5)	0.542
ALT (U/L)	25.0 (16.0, 41.0)	21.0 (14.0, 46.5)	25.0 (15.0, 42.0)	0.226
Glucose (mmol/L)	7.7 (6.0, 10.8)	8.1 (6.3, 11.6)	7.9 (6.1, 11.2)	0.267
Sodium (mmol/L)	140.0 (138.0, 142.0)	138.0 (135.0, 141.0)	140.0 (136.5, 142.0)	**<0.001**
Potassium (mmol/L)	4.5 (4.1, 4.8)	4.5 (4.1, 5.0)	4.5 (4.1, 4.8)	0.194
Chloride (mmol/L)	104.0 (101.0, 107.0)	100.0 (97.0, 104.0)	103.0 (99.0, 106.0)	**<0.001**
BUN (mmol/L)	8.3 (6.3, 12.3)	12.3 (8.9, 16.8)	9.6 (6.9, 14.4)	**<0.001**
Creatinine (µmol/L)	107.0 (86.0, 144.0)	131.5 (107.0, 164.0)	117.0 (90.5, 152.5)	**<0.001**
eGFR (ml/min/1.73 m^2^)	54.0 (36.1, 70.5)	38.4 (29.1, 52.1)	46.6 (32.3, 65.0)	**<0.001**
CK (U/L)	105.0 (65.0, 174.0)	78.0 (50.2, 147.5)	93.0 (58.0, 165.5)	**0.007**
LDH (U/L)	252.0 (217.0, 316.0)	283.0 (230.8, 372.2)	265.0 (218.5, 332.0)	**0.029**
hsTnI (ng/L)	39.0 (17.5, 136.5)	61.0 (30.0, 149.0)	46.0 (20.0, 143.2)	**0.039**
NT-proBNP (pg/mL)	5350.0 (3151.0, 10,691.0)	10733.0 (5486.5, 18,385.5)	6692.0 (3531.0, 14,395.5)	**<0.001**
CRP (mg/L)	10.3 (4.9, 21.9)	24.9 (6.4, 47.3)	12.2 (5.5, 33.1)	**<0.001**
IL-6 (pg/mL)	22.1 (11.3, 44.8)	40.6 (17.1, 79.6)	25.1 (12.9, 60.1)	**<0.001**
Fibrinogen (g/L)	4.0 (3.4, 4.7)	4.0 (3.1, 4.9)	4.0 (3.4, 4.8)	0.469
Erythrocytes (× 10^12^/L)	4.7 (4.4, 5.1)	4.4 (3.8, 4.9)	4.6 (4.2, 5.1)	**<0.001**
Hemoglobin (g/L)	138.0 (124.0, 150.0)	126.0 (111.0, 141.0)	134.0 (119.0, 148.0)	**<0.001**
pH	7.4 (7.3, 7.5)	7.4 (7.3, 7.4)	7.4 (7.3, 7.5)	0.709
pO_2_ (kPa)	8.8 (7.2, 10.4)	8.8 (7.3, 10.4)	8.8 (7.2, 10.4)	0.803
pCO_2_ (kPa)	5.2 (4.4, 6.3)	5.2 (4.5, 7.1)	5.2 (4.5, 6.4)	0.386
HCO_3_ (mmol/L)	23.9 (21.2, 27.0)	24.4 (21.3, 28.9)	23.9 (21.3, 27.4)	0.368

Data are presented as n (%), mean and standard deviation, or median and interquartile range (q1, q3). Differences between AHF patients who survived and those who died within 1 year after study inclusion were tested using Fisher’s exact test, t-test, or Mann–Whitney U test. The p-values <0.05 are considered significant and are depicted in bold. AF, atrial fibrillation; AHF, acute heart failure; ALT, alanine aminotransferase; apoB, apolipoprotein B-100; AST, aspartate aminotransferase; BMI, body mass index; BUN, blood urea nitrogen; CAD, coronary artery disease; CHF, chronic heart failure; CK, creatine kinase; CKD, chronic kidney disease; CMP, cardiomyopathy; COPD, chronic obstructive pulmonary disease; CRP, C-reactive protein; eGFR, estimated glomerular filtration rate; HDL-C, high-density lipoprotein cholesterol; HFrEF, heart failure with reduced ejection fraction; HFmrEF, heart failure with mildly reduced ejection fraction; HFpEF, heart failure with preserved ejection fraction; hsTnI, high-sensitivity troponin I; HDL-C, high-density lipoprotein cholesterol; JVD, jugular vein distension; LDH, lactate dehydrogenase; LDL-C, low-density lipoprotein cholesterol; LVEDd, left-ventricle end-diastolic diameter; LVEF, left-ventricular ejection fraction; MAP, mean arterial pressure; MetS, metabolic syndrome; NT-proBNP, N-terminal pro brain natriuretic peptide; NYHA, New York Heart Association Functional Classification; pO2, partial oxygen pressure; pCO2, partial carbon dioxide pressure; SPAP, systolic pulmonary artery pressure; TC, total cholesterol; T2DM, Diabetes Mellitus Type 2.

**Table 2:** Differences in serum levels and lipid content of VLDL parameters in AHF patients.

	Alive (*n* = 197)	Deceased (*n* = 117)	All (*n* = 314)	*p*-Value
VLDL-C (mg/dL)	12.7 (8.4, 20.2)	10.6 (7.7, 15.8)	11.8 (7.9, 19.1)	**0.036**
VLDL-TG (mg/dL)	39.7 (27.0, 65.7)	34.7 (25.8, 49.6)	37.3 (26.5, 58.4)	**0.021**
VLDL-PL (mg/dL)	11.6 (8.1, 18.3)	9.7 (7.1, 14.2)	10.8 (7.5, 17.0)	**0.018**
VLDL-apoB (mg/dL)	7.1 (5.0, 9.7)	6.5 (5.1, 8.8)	6.9 (5.0, 9.5)	0.402
VLDL-C/VLDL-apoB	1.9 (1.6, 2.2)	1.7 (1.5, 1.9)	1.8 (1.5, 2.1)	**<0.001**
VLDL-TG/VLDL-apoB	6.4 (5.1, 7.9)	5.4 (4.7, 6.3)	5.9 (5.0, 7.3)	**<0.001**
VLDL-PL/VLDL-apoB	1.8 (1.5, 2.2)	1.6 (1.3, 1.8)	1.7 (1.4, 2.1)	**<0.001**

Data are presented as median and interquartile range (q1, q3). Differences between AHF patients who were alive and those who died within 1 year after study inclusion were tested using the Mann–Whitney U test. Data were not available for one patient who died within 1 year after inclusion. The *p*-values <0.05 are considered significant and are depicted in bold. apoB, apolipoprotein B; C, cholesterol; VLDL, very-low-density lipoprotein; PL, phospholipid; TG, triglyceride.

**Table 3:** The ratios in various groups of AHF patients.

		VLDL-C/VLDL-apoB	VLDL-TG/VLDL-apoB	VLDL-PL/VLDL-apoB
T2D	no (*n* = 182)	1.71 (1.51, 2.08)	5.82 (4.83, 7.19)	1.66 (1.39, 2.03)
	yes (*n* = 132)	1.88 (1.56, 2.21)	6.10 (5.13, 7.35)	1.80 (1.49, 2.08)
		***p*** **= 0.012**	*p* = 0.349	*p* = 0.181
CAD	no (n = 158)	1.74 (1.51, 2.08)	5.62 (4.57, 6.89)	1.64 (1.41, 1.98)
	yes (*n* = 156)	1.82 (1.56, 2.20)	6.34 (5.13, 7.73)	1.82 (1.47, 2.16)
		*p* = 0.086	***p*** **= 0.002**	*p* = 0.025
AF	no (*n* = 144)	1.89 (1.60, 2.26)	6.53 (5.24, 8.12)	1.86 (1.54, 2.19)
	yes (*n* = 170)	1.71 (1.49, 1.98)	5.59 (4.73, 6.61)	1.59 (1.37, 1.95)
		***p*** **< 0.001**	***p*** **< 0.001**	***p*** **< 0.001**
Statins	no (*n* = 196)	1.78 (1.58, 2.15)	5.76 (4.60, 7.15)	1.67 (1.40, 2.02)
	yes (*n* = 118)	1.73 (1.48, 2.12)	6.31 (5.17, 7.42)	1.80 (1.49, 2.13)
		*p* = 0.296	***p*** **= 0.012**	*p* = 0.095
Venous overload *	no (*n* = 66)	2.13 (1.82, 2.39)	7.41 (5.97, 8.75)	2.05 (1.73, 2.25)
	yes (*n* = 248)	1.72 (1.50, 2.03)	5.70 (4.77, 7.03)	1.64 (1.39, 1.97)
		***p*** **< 0.001**	***p*** **< 0.001**	***p*** **< 0.001**
AHF type	New onset AHF (*n* = 27)	2.33 (2.08, 2.46)	8.04 (6.65, 9.51)	2.25 (1.96, 2.44)
	AHF following CHF (*n* = 287)	1.73 (1.51, 2.09)	5.79 (4.86, 7.14)	1.66 (1.41, 2.00)
		***p*** **< 0.001**	***p*** **< 0.001**	***p*** **< 0.001**

Data are presented as median and interquartile range (q1, q3). Differences in the ratios between the groups were tested using the Mann–Whitney U test. The *p*-values <0.017 are considered significant after a Bonferroni correction for multiple testing and are depicted in bold. AF, atrial fibrillation; AHF, acute heart failure; CAD, coronary artery disease; CHF, chronic heart failure; T2D, type 2 diabetes mellitus. * Any of the following: enlarged liver, peripheral edema, ascites, or jugular venous distension.

## Data Availability

Data are available within the article and Supplementary Materials.

## Supplementary Materials

The following supporting information can be downloaded at https://www.mdpi.com/article/10.3390/biom12101542/s1: Scheme S1. Study flowchart; Table S1. Chronic medication of AHF patients; Table S2. Univariable Cox regression analyses of parameters used for adjustment in the multivariable models; Table S3. Correlation analyses of VLDL-apoB with laboratory and clinical parameters.
