# Supplementary material for: Cholesterol Content of Very-Low-Density Lipoproteins Is Associated with 1-Year Mortality in Acute Heart Failure Patients
Source: Biomolecules. 2022 Oct 21;12(10):1542. doi: 10.3390/biom12101542 (PMC9599569; doi:10.3390/biom12101542)
Supplement: Supplementary file 1 [file biomolecules-12-01542-s001.zip › Table S1.pdf]

Table S1. Chronic medication of AHF patients

	**Alive (N=197)**	**Deceased (N=118)**	**All (N=315)**	**p-value**
Furosemide	105 (53.3%)	88 (74.6%)	193 (61.3%)	**< 0.001**
Chlortalidone	17 (8.6%)	9 (7.6%)	26 (8.3%)	0.835
Indapamide	12 (6.1%)	11 (9.3%)	23 (7.3%)	0.371
MRA	36 (18.3%)	31 (26.3%)	67 (21.3%)	0.117
Digoxin	14 (7.1%)	14 (11.9%)	28 (8.9%)	0.158
Nitrate	23 (11.7%)	16 (13.6%)	39 (12.4%)	0.724
Amiodarone	16 (8.1%)	9 (7.6%)	25 (7.9%)	1.000
Beta-blockers	119 (60.4%)	68 (57.6%)	187 (59.4%)	0.637
ACEI	101 (51.3%)	68 (57.6%)	169 (53.7%)	0.295
Ang II receptor antagonist	22 (11.2%)	11 (9.3%)	33 (10.5%)	0.705
Amlodipine	57 (28.9%)	34 (28.8%)	91 (28.9%)	1.000
Statins	76 (38.6%)	43 (36.4%)	119 (37.8%)	0.720
Insulin	28 (14.2%)	23 (19.5%)	51 (16.2%)	0.269
Metformin	27 (13.7%)	25 (21.2%)	52 (16.5%)	0.087
Sulphonylurea	23 (11.7%)	10 (8.5%)	33 (10.5%)	0.449
ASA	44 (22.3%)	39 (33.1%)	83 (26.3%)	**0.047**
Warfarine	48 (24.4%)	36 (30.5%)	84 (26.7%)	0.239

Data are presented as n (%). Differences between AHF patients who survived and those who died within 1 year after study inclusion were tested with the Fisher Exact test. P-values < 0.05 are considered significant and are depicted in bold.
ASA, Acetylsalicylic acid; ACEI, angiotensin converting enzyme inhibitor; MRA, mineralocorticoide receptor antagonist.
