# Supplementary material for: Cholesterol Content of Very-Low-Density Lipoproteins Is Associated with 1-Year Mortality in Acute Heart Failure Patients
Source: Biomolecules. 2022 Oct 21;12(10):1542. doi: 10.3390/biom12101542 (PMC9599569; doi:10.3390/biom12101542)
Supplement: Supplementary file 1 [file biomolecules-12-01542-s001.zip › Table S3.pdf]

Table S3. Correlation analyses of VLDL-apoB with laboratory and clinical parameters

	VLDL-apoB		
	r	p	N
Protein (g/L)	0.02	0.707	303
Albumin (g/L)	0.03	0.583	303
Fibrinogen (g/L)	0.25	**<0.001**	289
BUN (mmol/L)	0.06	0.274	313
eGFR	-0.06	0.252	314
CK (U/L)	0.05	0.339	314
NT-proBNP (pg/mL)	-0.01	0.901	314
CRP (mg/L)	0.10	0.082	314
IL-6 (pg/mL)	0.08	0.140	314
Hemoglobin (g/L)	0.05	0.355	314
MAP (mm Hg)	0.06	0.283	314

Data presented are the Spearman correlation coefficient r, the corresponding p-values, and the number of available samples (N).

P-values < 0.05 are considered significant and are depicted in bold.

BUN, blood urea nitrogen; CK, creatin kinase; CRP, C-reactive protein; eGFR, estimated glomerular filtration rate; IL-6, interleukin-6; MAP, mean arterial pressure; NT-proBNP, N-terminal pro brain natriuretic peptide.
