# Supplementary figures and images for: Cholesterol Content of Very-Low-Density Lipoproteins Is Associated with 1-Year Mortality in Acute Heart Failure Patients
Source: Biomolecules. 2022 Oct 21;12(10):1542. doi: 10.3390/biom12101542 (PMC9599569; doi:10.3390/biom12101542)

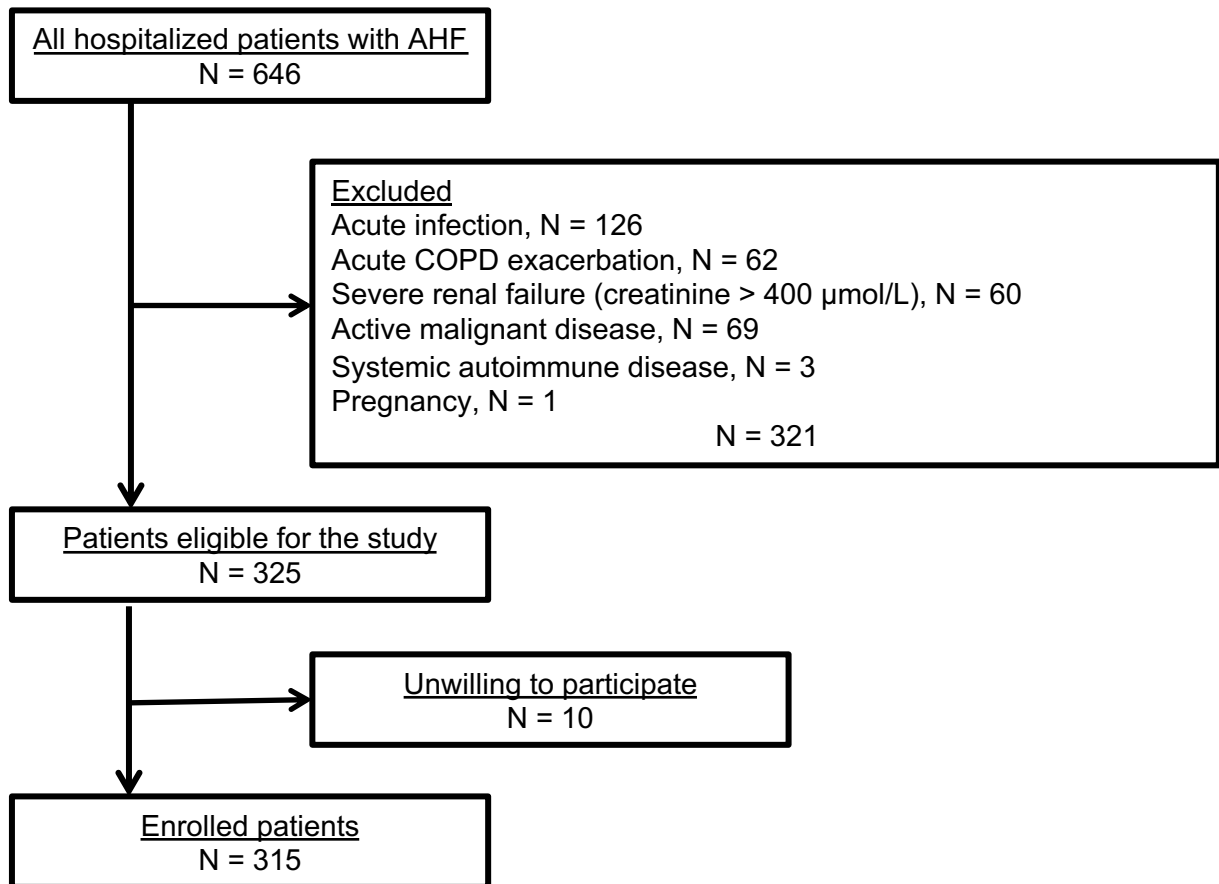

Supplement: Supplementary file 1 [file biomolecules-12-01542-s001.zip › Scheme S1.pdf]
